# Beyond the gut: the multisite microbiota and its emerging role in brain health

**DOI:** 10.1080/19490976.2026.2702091

**Published:** 2026-07-09

**Authors:** Seyedeh Marziyeh Jabbari Shiadeh, Mireia Valles-Colomer, Gregers Wegener, John Cryan, Carina Mallard, Maryam Ardalan

**Affiliations:** a Department of Physiology, Institute of Neuroscience and Physiology, Sahlgrenska Academy, University of Gothenburg, Gothenburg, Sweden; b Department of Medical and Life Sciences, Universitat Pompeu Fabra, Barcelona, Spain; c Department of Clinical Medicine, Translational Neuropsychiatry Unit, Aarhus University, Aarhus, Denmark; d APC Microbiome Ireland, University College Cork, Ireland; e Department of Anatomy and Neuroscience, University College Cork, Ireland

**Keywords:** Microbiota, brain, gut, neuroimmune signaling

## Abstract

The human microbiota encompasses diverse microbial communities inhabiting the gut, oral cavity, skin, respiratory tract, and urogenital system, each contributing uniquely to host physiology and health. While the gut microbiota has been extensively studied in the context of brain development and behavior through the microbiota–gut–brain axis, emerging evidence highlights that non-gut microbial ecosystems also shape neuroimmune processes and neurological outcomes. However, evidence supporting non-gut microbiota–brain interactions remains heterogeneous and is often indirect, with causal relationships largely unproven. A growing biological plausibility suggests that oral, skin, nasal, respiratory, and urogenital microbiota influence brain health through immune signaling, microbial metabolites, barrier integrity, and even direct neural pathways. This evidence is derived from a combination of human observational and epidemiological studies, complemented by mechanistic insights from animal models and in vitro systems. Microbial imbalance at these sites has been linked to neuroinflammation, neurodevelopmental alterations, and neurodegenerative diseases, although mechanistic insight remains limited. Moreover, cross-talk between different microbial niches may amplify or mitigate neurological effects, indicating the need for integrative, multi-site, and multi-omics approaches. By critically synthesizing evidence across multiple body sites, this mini-review highlights conceptual gaps, and the need to move beyond a gut-centric framework toward integrative, multisite investigations of microbiota–brain communication.

## Graphical abstract

**Figure:**
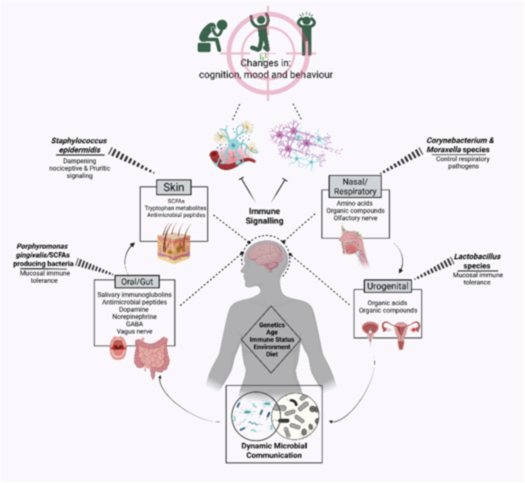

Multisite microbiota–brain interactions and their impact on cognition, mood, and behavior. Microbial communities across the gut, oral cavity, skin, respiratory tract, and urogenital system influence brain health through immune, metabolic, endocrine, and neural pathways. Microbial metabolites, pro- and anti-inflammatory signals, and community-derived factors including short-chain fatty acids (SCFAs), neurotransmitter-related metabolites, and immune-modulating molecules shape local and systemic physiology. Across sites, shifts in microbial composition affect barrier integrity, immune tone, and neuroactive compound production, ultimately converging on neural and immune pathways that regulate cognition, mood, and behavior. Host factors such as genetics, age, immune status, environment, and diet dynamically influence microbial composition, while cross-talk between different microbial niches may amplify or buffer neurological outcomes. The pathways shown represent proposed and biologically plausible routes of microbiota–brain communication. The strength of evidence supporting these interactions varies considerably across body sites, with some mechanisms being well established and others remaining largely hypothetical. Image is generated using Biorender software.

## Introduction

The human body is host to a vast and dynamic community of microorganisms collectively termed the microbiota that are located in diverse anatomical sites, including the gastrointestinal tract, skin, oral cavity, respiratory system, and urogenital tract.^1^ These microbial ecosystems are shaped by both environmental factors like diet, hygiene, climate, and pollutant exposure, and host-derived factors, including genetics, age, immune status, and epithelial barrier integrity.^2^ Among body sites, the gut microbiota is recognized as one of the most diverse and functionally rich ecosystems in the human body, having thousands of microbial species and an immense gene pool.^3^ In addition to the well-established influence of the gut microbiota on brain development, function, and disease, emerging evidence suggests that microbiota from other body sites also play significant roles in modulating neural processes and behavior.^4–8^ Microbial products and immune mediators from these communities can cross the blood–brain barrier or signal through peripheral nerves, thereby influencing brain homeostasis, including neuroimmune balance, synaptic function, neuronal excitability, and glial activity, and potentially contributing to neuroinflammation.^9–14^ Examples of these signals include microbial metabolites such as short-chain fatty acids (SCFAs), pro-inflammatory cytokines (e.g., IL-1β, IL-6, and TNF-*α*), and microbial components such as lipopolysaccharide, which can affect blood–brain barrier permeability and microglial activation.^11–14^ Current literature more often reports interactions of the gut and oral microbiomes with circulating immune mediators, whereas evidence for other body sites remains more limited.

Given that microbial communities impact the nervous system through diverse molecular layers including the genome, transcriptome, proteome, and metabolome, multi-omics approaches are crucial for comprehensively mapping these complex and site-specific interactions.^15^ ^,^ ^16^

The gut microbiota is a densely populated microbial ecosystem that plays a central role in brain development, function, and disease. Communication between the gut microbiota and the brain occurs through multiple, highly interconnected pathways, including immune, endocrine, and neural routes. These pathways involve microbial modulation of systemic and central immune responses, activation of neural circuits such as the vagus nerve and enteric nervous system, and the production of circulating microbial metabolites that can influence or cross the blood–brain barrier. Together, these mechanisms support a bidirectional gut–brain axis, in which microbial signals shape neurodevelopment and brain function, while brain-derived immune, neural, and hormonal signals reciprocally influence gut microbial composition.^17^ In certain pathological contexts, particularly neurodegenerative disorders, current evidence suggests a predominantly gut-to-brain influence, whereby microbial-derived factors contribute to neuroinflammation and disease progression. Through these mechanisms, gut-derived signals influence neurodevelopment, synaptic plasticity, and susceptibility to neuropsychiatric and neurodegenerative disorders.^18^ ^,^ ^19^

Gut microbes produce a wide range of neuroactive compounds including serotonin, dopamine, norepinephrine, and gamma-aminobutyric acid (GABA), as well as precursors and modulators of these neurotransmitters. Although many of these molecules act locally within the gut, they can also influence gut-to-brain signaling indirectly through immune activation, modulation of vagal afferents, and alterations in host neurotransmitter synthesis and metabolism, thereby affecting neurophysiology at both local and systemic levels.^20–22^ Emerging evidence suggests that these microbiota-derived neuroactive signals may also influence higher-order behavioral processes, including mood regulation, emotional reactivity, cognitive flexibility, and decision-making. Through interactions with neurotransmitter systems, immune pathways, and stress-response networks, alterations in gut microbial composition have been associated with behavioral and psychological outcomes, highlighting the potential therapeutic relevance of the gut microbiota in neuropsychiatric disorders.^23^

In parallel, microbial-derived metabolites such as SCFAs exert pleiotropic effects on host gene expression, immune maturation, and neuronal signaling, further highlighting the integrative nature of microbiota-driven communication with the brain.^11^ ^,^ ^12^ The production of these metabolites depends on the composition of the gut microbial community, which is typically dominated by the phyla *Bacteroidetes* and *Firmicutes*, including key genera such as *Bacteroides*, *Faecalibacterium*, and *Lactobacillus*. These taxa contribute to host health by producing SCFAs, such as butyrate, that support intestinal barrier integrity and modulate inflammatory responses.^24^

Beyond acute signaling, the gut microbiota can influence host gene expression programs relevant to brain function. Experimental studies, particularly in germ-free animal models, have demonstrated that microbial absence or disruption can alter neurotransmitter systems, synaptic plasticity, and behavioral outcomes, highlighting the importance of microbiota-derived signals during neurodevelopment.^19,25–29^ These effects are mediated in part through microbial metabolites, including SCFAs, which influence immune regulation, neuronal signaling, and gene expression.^30–32^ Collectively, these findings support the concept that gut microbial composition contributes to microbiota–brain communication through molecular, metabolic, and immune pathways.

While several recent reviews have begun to acknowledge microbiota–brain interactions beyond the gut, most remain either site-specific or implicitly gut-centric, with limited integration across microbial niches. This mini-review advances the field by systematically synthesizing evidence across multiple body sites including the oral cavity, skin, respiratory tract, and urogenital system and by explicitly considering how signals from these distinct microbial ecosystems may converge on shared neuroimmune and neural pathways. Rather than proposing a strictly hierarchical model dominated by a single microbial site, we propose a distributed network model of microbiota–brain communication, in which parallel and context-dependent signaling from multiple peripheral niches collectively shapes neuroimmune balance and brain health. In this framework, interactions between microbial sites may amplify, buffer, or modify neurological outcomes, depending on developmental stage, host vulnerability, and environmental exposures. By adopting this integrative perspective, the review highlights key conceptual gaps and emphasizes the need for multi-site, multi-omics, and longitudinal approaches to better define causal mechanisms and therapeutic opportunities in microbiota–brain research.

## The oral microbiome: gateway to systemic and neural health

Beyond the gut, the oral microbiome supports host immunity by regulating both innate and adaptive immune responses, primarily through interactions at mucosal surfaces.^33^ Under physiological conditions, oral microbial communities contribute to colonization resistance against pathogens, regulation of local pH, maintenance of mucosal barrier integrity, and the modulation of the host immune system. Through continuous interaction with saliva, epithelial surfaces, and immune cells, the oral microbiota helps preserve a balanced inflammatory state that is critical for both oral and overall health.^34^ ^,^ ^35^

Evidence from human studies indicates that commensal microorganisms stimulate the production of salivary immunoglobulins such as IgA together with antimicrobial peptides, which help maintain microbial homeostasis and prevent pathogenic overgrowth.^36^ Although the oral microbiome is relatively stable at the community level in health, oral dysbiosis and periodontitis are strongly linked to increased systemic inflammatory burden and neuroinflammation. Alzheimer’s disease has received the most attention, with numerous epidemiological studies, together with mechanistic evidence largely derived from animal and in vitro models, associating periodontal disease and specific oral pathogens with systemic inflammation, blood–brain barrier disruption, and Alzheimer’s disease pathology. However, emerging evidence indicates that alterations in the oral microbiome also occur in other brain‑related disorders, including schizophrenia, Parkinson’s disease and other neuropsychiatric conditions, where distinct oral microbial signatures and dysbiosis have been reported.^8,37–39^ Several recent case–control studies indicate that individuals with schizophrenia exhibit a dysbiotic oral microbiome compared with healthy controls. Salivary and oropharyngeal profiling reveals broad shifts in community structure, including altered α‑diversity, enrichment of lactic acid bacteria (such as *Lactobacillus* and Bifidobacterium), and depletion of commensal genera *like Neisseria, Haemophilus and Capnocytophaga*, with some taxa differentiating patients from controls with high accuracy. Importantly, these microbial changes are already detectable in drug‑naïve, first‑episode schizophrenia and correlate with symptom severity and peripheral inflammatory markers. This supports a possible association with disease-related biological processes rather than being solely a consequence of long‑term antipsychotic treatment or poor oral hygiene.^40–42^

The stability and composition of the oral microbiota can vary substantially depending on the sampling location such as saliva, tongue dorsum, or subgingival plaque, which may influence observed associations and complicate cross-study comparisons.^43^ Associative evidence from human cohorts suggests that dysbiosis at specific oral niches is linked to elevated systemic levels of pro-inflammatory cytokines, including IL-1 and TNF-*α*, which are known contributors to chronic inflammation and have been associated with cognitive decline and neurodegeneration.^13^ ^,^ ^14^ However, these findings remain largely correlative, and direct causal relationships between oral microbial changes and brain dysfunction have yet to be established.

Several studies, primarily in animal models and limited human post-mortem analyzes, have reported the detection of periodontal pathogens, including *Porphyromonas gingivalis, Prevotella,* and *Fusobacterium*, in the bloodstream and in some cases, in brain tissue, raising the possibility of direct effects on neural health.^14^ ^,^ ^44^ While these observations raise the possibility of direct microbial or microbial product access to the central nervous system, the presence of intact bacteria within brain tissue remains controversial. Alternative explanations include transient bacteremia, contamination, or detection of bacterial components rather than viable organisms.

In addition, interpretation of associations between oral dysbiosis and neurological outcomes must consider important confounding factors, including age, comorbid medical conditions, medication use, and socioeconomic status. These factors strongly influence oral health, access to dental care, and systemic inflammatory burden, and may independently contribute to neuropsychiatric and neurodegenerative risk, complicating causal inference in human cohort studies.^45^ ^,^ ^46^

Accordingly, maintaining oral microbial homeostasis is essential for preventing systemic and neuroinflammatory diseases. Interest in microbiome‑modulating approaches such as probiotics and prebiotics is growing, but current clinical data are heterogeneous, strain‑specific, and often underpowered, so no standardized recommendations for ‘brain‑targeted’ oral probiotics can yet be made.^47^ Well-controlled longitudinal and interventional studies are therefore required to determine whether modulation of the oral microbiome can causally influence brain health or neuropsychiatric outcomes.

## Neuroimmune interactions at the skin surface

The skin microbiota, a diverse community of microorganisms, is shaped by a complex interplay of environmental and host-derived factors. Evidence from human observational and cohort studies indicates that environmental influences such as climate, hygiene practices, and exposure to pollutants, as well as host factors including genetics, age, immune status, and the integrity of the skin barrier, all contribute to the composition and stability of the skin microbiota.^2^ This dynamic microbial community is not only essential for maintaining skin homeostasis but also plays a pivotal role in modulating neuroimmune interactions at the skin surface. Recent studies, primarily in animal models and ex vivo human skin systems, have revealed that the skin microbiota communicates bidirectionally with both peripheral nerves and resident immune cells, thereby influencing neuroimmune responses and contributing to the pathogenesis of several dermatological conditions with neurological components.^48–50^ For example, preclinical studies in mice have shown that certain commensal bacteria, such as *Staphylococcus epidermidis* strains, produce metabolites that can modulate the activity of cutaneous sensory neurons, potentially dampening nociceptive and pruritic signaling.^51^ *Staphylococcus epidermidis* has also been implicated, primarily through animal studies and limited translational evidence, in early neuroimmune regulation relevant to preterm birth and subsequent neurodevelopmental vulnerability.^52^ While these findings suggest a broader role for skin-associated microbes in early-life neuroimmune programming, direct causal evidence in humans remains limited.

Microbial-derived molecules may also interact with skin-resident immune cells, such as Langerhans cells, dendritic cells, and mast cells. Mechanistic insights into these interactions are largely derived from animal models and in vitro studies, which demonstrate effects on local immune activation, immune tolerance, and inflammatory signaling.^53^ In parallel, human clinical studies indicate that the integrity of the skin barrier, which is influenced by both host genetics and microbial composition, plays a crucial role in regulating neuroimmune balance. Disruption of barrier function can facilitate the penetration of microbial products and environmental allergens, further activating cutaneous nerves and immune cells and perpetuating a cycle of inflammation and neurological symptoms.^54^

Importantly, cutaneous neuroimmune activation may influence central nervous system function through several indirect but biologically plausible pathways, including sustained release of pro-inflammatory cytokines into the circulation, activation of sensory afferent pathways projecting to stress- and emotion-related brain regions, and modulation of hypothalamic–pituitary–adrenal (HPA) axis activity. These mechanisms provide a potential link between skin-localized microbial dysbiosis and broader central neuroimmune changes, including altered microglial activation and blood–brain barrier integrity, particularly during sensitive developmental windows.^55^ ^,^ ^56^

Early-life colonization patterns further shape the development of the cutaneous microbiome and its neuroimmune functions. In vaginally delivered infants, exposure to maternal vaginal and perineal microbiota, including *Lactobacillus*-dominated communities, seeds the neonatal skin and mucosal surfaces and promotes the establishment of commensal ecosystems that support barrier maturation and immune education. In contrast, cesarean delivery is associated with reduced exposure to maternal vaginal microbes and relatively greater colonization by skin- and environment-derived taxa. These altered colonization trajectories have been linked to dysregulated immune development and an increased risk of immune-mediated conditions in childhood. These observations suggest that maternal skin and vaginal microbiota act as critical reservoirs for the initial cutaneous microbiome of the next generation, with potential long‑term consequences for skin barrier function, inflammatory set‑points, and neuroimmune signaling along the skin–brain axis.^57^ ^,^ ^58^

Emerging integrative frameworks, such as the recently proposed ‘skin–immune–neuro–gastro–endocrine (SINGE) system,’ has been proposed as an integrative conceptual model describing the skin as a neuroendocrine interface that coordinates interactions between barrier function, immune signaling, microbial communities, and neuroendocrine pathways.^59^ Within this framework, barrier disruption and microbial dysbiosis may trigger immune and neural cascades that extend to systemic and central inflammation. Although direct evidence linking specific skin microbiome signatures to defined psychiatric or neurodegenerative disorders remains limited, converging data from atopic dermatitis, chronic pruritic conditions, and stress-related skin diseases support a bidirectional skin–brain axis. In this context, cutaneous dysbiosis and neuroimmune activation can exacerbate anxiety, depression, and itch–scratch cycles, while also being modulated by psychological stress. Together, these findings suggest that, alongside the gut and oral microbiomes, the skin microbiota may form part of a distributed peripheral network capable of shaping neuroimmune balance and influencing vulnerability to brain disorders.^59^ ^,^ ^60^ Continued investigation of the cutaneous microbiome–neuroimmune axis may reveal novel therapeutic targets across dermatological and neurological conditions.

## The airway microbiome and the olfactory route to the brain

While the nasal and respiratory tract microbiota are so far less well characterized, they are increasingly recognized as key modulators of neurological health, exerting their influence through both immune-mediated and direct anatomical pathways. Human observational studies indicate that the upper airway, particularly the nasal cavity, includes a unique microbial community that interacts closely with the mucosal immune system and the olfactory neuroepithelium. The nasal microbiota is generally dominated by *Corynebacterium*, *Staphylococcus*, and *Moraxella* species, which help protect the respiratory epithelium by modulating immune responses and limiting overgrowth of potential respiratory pathogens.^61^

Alterations in nasal microbiota composition have been associated with olfactory dysfunction and have been reported in human cohorts and animal models of neurodevelopmental and neurodegenerative disorders.^50^ ^,^ ^62^ One proposed mechanism involves the modulation of immune signaling. Evidence from both preclinical models and clinical studies suggests that the respiratory tract microbiota can influence local and systemic immune responses by regulating the production of cytokines, chemokines, and neuroactive microbial metabolites such as SCFAs. These immune mediators can cross the blood-brain barrier or signal through peripheral nerves, thereby affecting brain homeostasis and potentially contributing to neuroinflammation.^63^ ^,^ ^64^ Another critical pathway for the nasal and respiratory tract microbiota is the direct anatomical connection between the nasal cavity and the brain via the olfactory nerve. This route provides a potential conduit for microbial components, toxins, or inflammatory mediators to bypass the blood-brain barrier and access the CNS directly. Both animal studies and clinical observations have demonstrated that certain pathogens and microbial products can be transported along the olfactory nerve, leading to localized neuroinflammation and, in some cases, contributing to the pathogenesis of neurodegenerative conditions.^62^ However, although the olfactory route represents a biologically plausible mechanism linking the airway microbiota to the CNS, current evidence does not support a uniform or broadly applicable role across all neurodegenerative disorders, and its clinical relevance likely varies depending on disease context and host susceptibility. Furthermore, disruptions in the pulmonary microbiota may alter the permeability of both the lung-blood and blood-brain barriers, facilitating the translocation of microbial products and inflammatory molecules into the CNS.^65^ Therefore, the interplay between the airway microbiota, immune signaling, and direct neural pathways offers novel insights into the etiology of neurodevelopmental and neurodegenerative disorders, and indicates the potential for microbiome-targeted interventions in the prevention and treatment of CNS diseases. A critical consideration is whether airway microbiota changes are a cause or consequence of neurological disease, particularly in aging and neurodegeneration. Cross-sectional studies report nasal dysbiosis correlating with olfactory loss and neuroinflammation in Alzheimer’s disease and Parkinson’s disease, but longitudinal data and animal models suggest early microbial shifts may precede pathology via olfactory invasion, while neurodegeneration could secondarily impair nasal immunity and epithelium. Bidirectional interactions likely amplify risk over time, highlighting the need for prospective studies to clarify causality.^66^ ^,^ ^67^

## The urogenital microbiome and hormone–brain interactions

The urogenital tract, particularly the vaginal microbiome, contains a complex and dynamic microbial ecosystem that is increasingly recognized as a potential contributor to brain health through its effects on systemic immunity, endocrine signaling, and reproductive physiology.^68–70^ Through interactions with host immune and hormonal pathways, the urogenital microbiome may influence neurodevelopment, mood regulation, and susceptibility to inflammatory and neuropsychiatric conditions. Evidence from human clinical and epidemiological studies indicates that, although the urogenital microbiome is less extensively characterized than the gut or oral microbiota, especially in terms of microbial diversity and host interactions, it has garnered growing attention in recent years for its role in host health and disease susceptibility.^1^ In females, the vaginal microbiota represents the well-characterized microbial component of the urogenital tract. It is typically dominated by *Lactobacillus* species (e.g., *L. crispatus, L. jensenii*), which contribute to maintaining an acidic environment (pH < 4.5) through lactic acid production. This acidic environment inhibits the growth of pathogens, supports mucosal immune tolerance, and modulates local cytokine and chemokine profiles, thereby shaping both innate and adaptive immune responses.^71^

Disruption of this microbial balance, a condition referred to as bacterial vaginosis (BV), is associated with increased susceptibility to sexually transmitted infections, pelvic inflammatory disease, preterm birth, and even increased risk of infertility and adverse pregnancy outcomes.^72–74^ Beyond these local consequences, emerging human and preclinical evidence suggests that urogenital dysbiosis may contribute to systemic inflammation and altered immune function, for example by increasing epithelial permeability, facilitating translocation of microbial products, and altering systemic cytokine and acute‑phase responses. Microbial metabolites such as SCFAs, as well as pathogen-associated molecular patterns (PAMPs), can influence distant organ systems via circulation and have been implicated in chronic inflammatory diseases.^75^ However, direct causal links between urogenital microbial alterations and brain dysfunction in humans remain limited, and current evidence is largely inferential, based on associations between chronic urogenital inflammation, systemic immune activation, and conditions such as depression, cognitive decline, and adverse neurodevelopment in offspring.

Vaginal and urinary tract dysbiosis may affect neurological function through systemic inflammatory signaling or by altering neuroendocrine pathways, particularly during pregnancy and menopause when hormonal regulation significantly impacts microbial composition.^76^ The estrobolome a defined set of microbial enzymes (notably *β*-glucuronidases) that deconjugate and reactivate circulating estrogens modulates the amount of active hormone available to host tissues. In this context, microbial metabolism of sex hormones represents a potential route for microbiome-to-brain communication. By regulating the bioavailability and recirculation of estrogens, estrobolome-associated microbial activity may influence immune responses, neuroendocrine signaling, and physiological processes relevant to mood, cognition, and neurodevelopment. Fluctuations in sex hormone levels, in turn, reshape the vaginal and urinary microbiota, suggesting a bidirectional hormone–microbiome–brain axis.^77^

The urinary tract, once considered sterile, is now known to contain a distinct microbial community termed the “urinary microbiota.” Advances in sequencing-based methods have revealed consistent presence of non-pathogenic bacterial taxa like *Corynebacterium*, *Streptococcu*s, *Lactobacillu*s, and *Gardnerella* in the urinary tract of healthy individuals.^78–80^ Alterations in this community have been linked to recurrent urinary tract infections, overactive bladder, interstitial cystitis, and some forms of chronic pelvic pain, all of which are associated with increased systemic inflammation, altered autonomic tone, and a high burden of anxiety and depressive symptoms. Importantly, the relevance of the urogenital microbiome to brain health is likely sex-specific. In females, vaginal microbial composition and hormone–microbiome interactions are closely linked to reproductive state, pregnancy, and menopause, with potential implications for neurodevelopmental and mood-related outcomes.^81^ In males, although the urogenital microbiome is less well characterized, emerging evidence suggests that urinary and prostate-associated microbial communities, together with androgen-mediated immune regulation, may influence systemic inflammation and stress-related or pain-related neurological outcomes, highlighting the need for sex-specific investigation.^82–84^

Moreover, the urogenital microbiota may interact bidirectionally with other microbial systems, such as the gut microbiome, through the so-called gut–bladder axis. Cross‑seeding, microbial migration, or shared systemic immune and endocrine signaling between these niches can influence host susceptibility to infection, inflammatory disease, and potentially stress‑related and pain‑related brain disorders.^85^

## Conclusions & future directions

Recent longitudinal and multi-omics studies have revealed that distinct body sites contain unique microbial communities that interact with the host immune and nervous systems in different ways.^1^ ^,^ ^8^ Together, the evidence reviewed here supports the concept that microbiota–brain communication extends beyond the gut and involves multiple peripheral microbial ecosystems, including the oral cavity, skin, respiratory tract, and urogenital tract. These microbial communities should therefore be viewed not as isolated entities but as components of a coordinated host-level ecosystem capable of influencing neuroimmune homeostasis through immune, metabolic, endocrine, and neural pathways. At the same time, important uncertainties remain. Available studies more commonly examine associations between the gut and oral microbiomes and circulating cytokines, whereas evidence for the skin, respiratory, and urogenital microbiomes remains comparatively limited. The evidence available across microbial niches is heterogeneous, reflecting differences in sampling approaches, analytical methodologies, and study designs. In addition, while bacteria dominate most studied body sites, the relative contributions of viruses, archaea, and fungi to microbiota–brain communication remain poorly understood. It is also not yet clear whether interactions among microbial communities at different body sites amplify, buffer, or otherwise modify neurological outcomes through shared immune and metabolic pathways. Furthermore, host-related factors such as age, diet, medication use, environmental exposures, and underlying health conditions may influence microbiota–brain interactions and should be carefully considered in future investigations.

Addressing these knowledge gaps will require integrative, multi-site, and multi-omics approaches that combine longitudinal human studies with mechanistic animal models. Future investigations should examine how microbial communities from different body sites interact with one another and with the nervous system, identify key metabolites and signaling molecules involved in microbiota–brain communication, and determine their contributions to neurodevelopment, behavior, and disease susceptibility.
